# Activation of Hydrogen Peroxide to Peroxytetradecanoic Acid Is Responsible for Potent Inhibition of Protein Tyrosine Phosphatase CD45

**DOI:** 10.1371/journal.pone.0052495

**Published:** 2012-12-27

**Authors:** Alicja Kuban-Jankowska, Jack A. Tuszynski, Philip Winter, Magdalena Gorska, Narcyz Knap, Michal Wozniak

**Affiliations:** 1 Department of Medical Chemistry, Medical University of Gdansk, Gdansk, Poland; 2 Department of Oncology, University of Alberta, Edmonton, Alberta, Canada; 3 College of Health, Beauty Care and Education in Poznan, Faculty in Gdynia, Gdynia, Poland; University of Tennessee, United States of America

## Abstract

Hydrogen peroxide induces oxidation and consequently inactivation of many protein tyrosine phosphatases. It was found that hydrogen peroxide, in the presence of carboxylic acids, was efficiently activated to form even more potent oxidant - peroxy acid. We have found that peroxytetradecanoic acid decreases the enzymatic activity of CD45 phosphatase significantly more than hydrogen peroxide. Our molecular docking computational analysis suggests that peroxytetradecanoic acid has a higher binding affinity to the catalytic center of CD45 than hydrogen peroxide.

## Introduction

Protein tyrosine phosphatases (PTPs) are enzymes that dephosphorylate proteins on tyrosine residues and together with protein tyrosine kinases are responsible for the regulation of signaling events that control fundamental biological processes [1]. A hallmark of the classical PTP enzymes is the strictly conserved primary structure including cysteine and arginine residues in the catalytic domain which constitutes a phosphate-binding pocket [2]. The cysteine residue localized in the catalytic center exists in a thiolate anion form and is susceptible to oxidation. Oxidation of the cysteine residue, leading to inactivation of the enzyme, contributes to the reversible formation of sulfenic acid residue. Highly oxidizing conditions can further induce oxidation to the sulfinic and sulfonic acid residues, which is considered irreversible under physiological conditions [3].

Hydrogen peroxide is an endogenous signaling agent and can regulate the activity of protein tyrosine phosphatases via oxidation of the catalytic cysteine residue. Inactivation of these enzymes by hydrogen peroxide can be reversed by cellular reducing agents such as glutathione [4]. Hydrogen peroxide in the presence of carboxylic acids can transform into a more potent oxidant – a respective peroxy acid [5]. This reaction can occur spontaneously or can be catalyzed by certain enzymes. It was demonstrated that lipases catalyzed the synthesis of peroxytetradecanoic acid from hydrogen peroxide and tetradecanoic acid (myristic acid) [6]. Myristic acid plays a role in protein modification. Myristoylation is an irreversible, co-translational protein modification essential for membrane targeting and signal transduction. Having undergone peroxidation, myristic acid might function as a regulator of phosphatase activity and thus affect many biological processes in the cell.

Protein tyrosine phosphatase CD45 is one of the key regulatory enzymes abundantly expressed in leukocytes. Interestingly, similar level of CD45 expression has been detected in pancreatic acinar cells. CD45 phosphatase negatively regulates cytokine production, thus the decrease in CD45 activity may be implicated in the pathogenesis of acute pancreatitis (AP) [7]. Pancreatic acinar cells were found to respond to pancreatitis-associated ascitic fluid producing pro-inflammatory cytokines, e.g. TNF alpha, which indicates potential association of pancreatic CD45 down-regulation and the progression of AP [8].

Due to the fact that peroxy acids are strong oxidants and may inactivate the PTPs via oxidation of catalytic center thiolate, we decided to investigate whether peroxytetradecanoic acid would have an effect on phosphatase CD45 activity [9]. We have investigated and compared the effects of treatment with hydrogen peroxide and peroxytetradecanoic acid on the enzymatic activity of recombinant CD45. Moreover, to gain a better insight into molecular mechanisms of action, we performed molecular docking computational analysis to study the binding affinity of hydrogen peroxide and peroxytetradecanoic acid to the catalytic center of CD45.

## Materials and Methods

### 1. Peroxytetradecanoic Acid Synthesis

Peroxytertradecanoic acid was chemically synthesized, using Parker’s method [9] in the reaction of tetradecanoic acid with hydrogen peroxide (from Sigma). The purity of the prepared compound was tested with NMR and IR spectroscopy. Peroxytetradecanoic acid in a powder form was stored in −80°C.

### 2. Recombinant PTP CD45 Activity Assay

Human recombinant protein tyrosine phosphatase CD45 expressed in a Baculovirus Sf9 expression system was obtained from Calbiochem (San Diego, CA). The assay was performed on 96-well microplates precoated with albumin, as described previously [10]. The working concentration of CD45 in tested samples was 130 nM (10 µg/mL). The enzyme was incubated with different concentrations of hydrogen peroxide, tetradecanoic acid, and peroxytetradecanoic acid. The solution of the enzyme in Tris buffer (pH 7.4) was used as a control. All samples were incubated for 15 min at 37°C. Then 1 mM chromogenic substrate *para*-nitrophenyl phosphate (*p*NPP) (Sigma) dissolved in 50 mM Tris buffer, pH 7.4 was added. After the following 5 min of incubation, PTP activity was measured. Active PTP hydrolyzed *p*NPP to *p*-nitrophenol and inorganic phosphate. The production of yellow colored *p*-nitrophenol was assessed as an increase in absorbance at 405 nm using microplate reader Jupiter (Biogenet) and DigiRead Communication Software (Asys Hitech GmbH). Subsequently 10 mM dithiothreitol (DTT) (Sigma) was added to each sample for 30 minutes and a potential recovery of the enzymatic activity was assessed using the same technique.

### 3. Computer Simulations with Docking Software

#### 3.1. Receptor and ligands preparation

The 1YGU PDB file was downloaded from the Protein Data Bank (http://www.rcsb.org/). 1YGU includes two domains of CD45: the D1 domain (which contains the PTP active site) and the D2 domain (which contains an inactive pseudo-phosphatase domain). The chain A residues 608–890 were extracted, which corresponds to the D1 domain. In order to correct for amino acid modifications in the 1YGU structure, the UniProtKB sequence P08575, a reference protein sequence for the longest isoform of human CD45 (PTPRC gene), was downloaded from the UniProt database (http://www.uniprot.org/). The sequence region 633–915 was extracted, which again corresponds to the D1 domain. The SWISS-MODEL web server (http://swissmodel.expasy.org/) [11] was used to generate a modeled structure using the 1YGU residues as the template structure and the equivalent P08575 region as the target sequence. This resulted in the catalytic cysteine being restored, and the selenomethionine residues being restored to methionine. The QMEAN Z-Score, which is an estimate of the quality of the model, was reported by SWISS-MODEL to be –1.78 [12]. Subsequently, hydrogen atoms were added to the modeled structure using the PDB2PQR webserver version 1.8 (http://kryptonite.nbcr.net/pdb2pqr/) [13]. The titratable residue states were specified with PDB2PQR in order to ensure that these residues matched the expected states during the first step of the phosphatase reaction mechanism [20]. The structures of each ligand were either drawn in ChemDraw (http://www.cambridgesoft.com/) or downloaded in 2-dimensional SDF format from PubChem (http://pubchem.ncbi.nlm.nih.gov/). The phosphotyrosine peptide was extracted from the 1YGU PDB structure. The ligands were processed with Schrodinger LigPrep version 25111 (http://www.schrodinger.com/). The default LigPrep options were used. LigPrep added hydrogens and generated initial 3-dimensional conformations for each of the ligands.

#### 3.2. Molecular docking

AutoDockTools version 1.5.4 [14] was used to convert the receptor and ligand library to PDBQT format. The molecular docking program AutoDock Vina version 1.1.1 [15] was used to perform the docking. A binding box was defined to be centered at the position of the phosphorus atom in the phosphotyrosine peptide in the bound complex, and the dimensions of the box were defined to be a cube with length of 25 Å, to allow for the largest ligands to assume fully extended conformations. Docking with a fully rigid receptor was performed. The Vina parameter exhaustiveness increases the time spent on the search; for the final docking runs an exhaustiveness parameter of 128 was used. Preliminary docking runs supported that this parameter was more than sufficient; increasing the exhaustiveness is not expected to significantly change the affinity scores. A set of 6 repetitions were performed with different random seeds. Since the search of ligand performed by Vina is stochastic, the results will be different depending on the random seed. The reported binding affinities are the means of the repetitions and the reported errors are the standard deviations. To select the best pose for each ligand, the poses generated by Vina were clustered with 1.5 Å RMSD thresholds, and the pose with strongest affinity in the largest cluster of poses was selected.

### 4. Statistical Analysis

The non-computational experiments were performed at least three times. The results were expressed as mean ± S.E.M. Statistical analyses were performed using the combination of ANOVA and Tukey’s test (GraphPad Prism Software v.4). Differences between the means were considered significant for p<0.05.

## Results and Discussion

**Figure 1:**
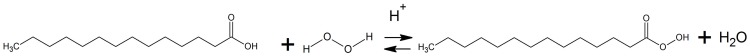
Synthesis of peroxytetradecanoic acid. The peroxytetradecanoic acid is formed in the reaction of tetradecanoic acid with hydrogen peroxide.

It is known that protein tyrosine phosphatases, due to the thiolate anion in the active site, are highly sensitive to oxidation and may be inactivated by hydrogen peroxide [16]. Hydrogen peroxide in the presence of carboxylic acids may be activated spontaneously, e.g. formic acid [17] or by enzymes to form the respective peroxy acids (Fig. 1.). It has been shown that the αβ-hydrolase family of enzymes, including lipases, display unexpectedly diverse catalytic activities: hydrolytic as well as perhydrolytic [18]. This second activity is responsible for transformation of hydrogen peroxide into peroxycarboxylic derivatives in the presence of carboxylic acids [19]. Peroxycarboxylic acids are considered to be the most potent oxidants of all organic peroxides, mainly since they possess the highly reactive peroxycarboxyl group [5]. The lipase-catalyzed formation of peroxytetradecanoic acid from hydrogen peroxide and tetradecanoic acid has been previously reported [6]. Based on colocalization of lipase and protein tyrosine phosphatase CD45 in pancreatic cells, we suggest that peroxy acids may be unexpected potential regulators of CD45 activity.

**Figure 2:**
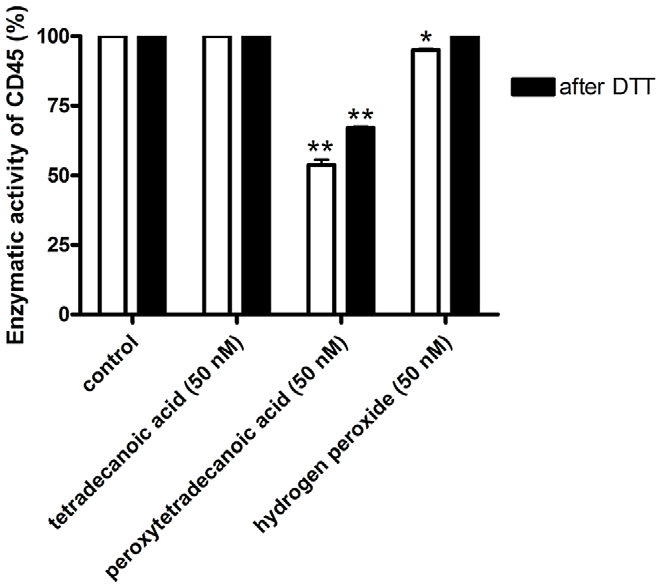
The enzymatic activity of PTP CD45. The effect of tetradecanoic acid, peroxytetradecanoic acid and H_2_O_2_ on enzymatic activity of PTP CD45 before and after treatment with DTT. The enzyme CD45 (130 nM) was incubated with 50 nM tetradecanoic acid, peroxytetradecanoic acid and H_2_O_2_ at 37°C for 15 minutes. The activity was measured after adding 1 mM pNPP in 50 mM Tris buffer, pH 7.4. After the following 5 min of incubation, PTP activity was measured using microplate reader at 405 nm. Subsequently 20 mM dithiothreitol (DTT) was added to each sample for 30 minutes and a potential recovery of the enzymatic activity was assessed using the same technique. The results were expressed as percent of activity of control sample in Tris buffer. The data from three independent experiments were present as mean ± S.E.M; * significantly different (P<0.05) from control, ** significantly different (P<0.001) from control. The data were analysed using the combination of ANOVA and Tukey’s test (GraphPad Prism Software v.4).

We examined the inhibitory effect of hydrogen peroxide and peroxidized tetradecanoic acid on the enzymatic activity of CD45. Our findings show that peroxytetradecanoic acid is a more potent inhibitor of CD45 than hydrogen peroxide (Fig. 2). A physiologically relevant concentration of 50 nM peroxytetradecanoic acid causes a 50% decrease of CD45 enzymatic activity, while treatment with the same concentration of hydrogen peroxide causes only about a 5% loss of the activity.

**Figure 3:**
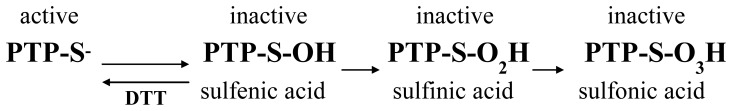
Oxidation steps of PTP catalytic cysteine residue. The cysteine residue exists in a thiolate anion form and may undergo oxidation to the inactive sulfenic acid residue form. This conversion is reversible, but highly oxidizing conditions can further induce oxidation to the sulfinic and sulfonic acid residues, which is considered irreversible under physiological conditions.

The catalytic cysteine residue of active PTP CD45 exists in a thiolate anion form and can be reversibly oxidized to sulfenic acid residue (SOH), or it may undergo irreversible oxidation to form sulfinic (SO_2_H) and sulfonic acid (SO_3_H) residues (Fig. 3) [3]. Our results show that the inactivation of CD45 by 50 nM hydrogen peroxide can be completely reversed by treatment with the reducing agent dithiothreitol (DTT), which suggests that the reaction of hydrogen peroxide with the catalytic cysteine residue of CD45 yields sulfenic rather than sulfinic or sulfonic residues. In contrast, treatment of the enzyme inactivated by 50 nM peroxytetradecanoic acid with DTT yields only a return to 67% of the initial enzymatic activity (Fig. 2). This suggests that peroxytetradecanoic acid, being a more potent oxidant than hydrogen peroxide, predominantly converts the active site cysteine of CD45 to the sulfinic or sulfonic acid residues (Fig. 3). The observation that tetradecanoic acid, without peroxycarboxyl group (COOOH), had no inhibition of enzymatic activity of CD45 supports this conclusion.

We performed molecular docking to investigate the binding energy (affinity) and binding conformations (poses) of peroxytetradecanoic acid and hydrogen peroxide to the catalytic site of CD45. For molecular docking we used the program AutoDock Vina in order to search through the possible poses of ligands in a given binding site and to determine the most likely pose based on an energy scoring function. AutoDock Vina is a new docking program based on AutoDock [14]. AutoDock Vina was selected as the docking software for this study due to its high performance and improved accuracy as compared with AutoDock [15]. The receptor structure used was the phosphatase active site of CD45, based on the PDB structure 1YGU, which is an X-ray diffraction structure of the CD45 cytosolic domains with a resolution of 2.9 Å, including a bound phosphotyrosine peptide [20]. The results of the docking calculated peroxytetradecanoic acid to have stronger binding affinity, –5.5 kcal/mol, than hydrogen peroxide, –3.1 kcal/mol; for comparison the binding affinity for a phosphotyrosine residue was calculated to be –6.3 kcal/mol (Table 1). The molecular docking results show the increased binding affinity of peroxytetradecanoic acid relative to hydrogen peroxide, which may partially explain the stronger inhibitory effect observed in the experimental results.

**Table 1:** The calculated binding affinities to CD45 catalytic center.

Ligand name	Chemical formula of ligand	Binding affinity (kcal/mol)
Phosphotyrosine	pTyr	−6.3
Peroxytetradecanoic acid	CH_3_(CH_2_)_12_COOOH	−5.5
Hydrogen peroxide	H_2_O_2_	−3.1

The binding affinity of the peroxytetradecanoic acid, hydrogen peroxide and phosphotyrosine (natural substrate as a control) calculated with docking software AutoDock Vina version 1.1.1. The receptor structure used for affinity calculations was based on the PDB structure 1YGU and ligands were drawn in ChemDraw and processed with Schrodinger LigPrep version 25111. The data present the means of 6 repetitions with different random seeds.

**Figure 4:**
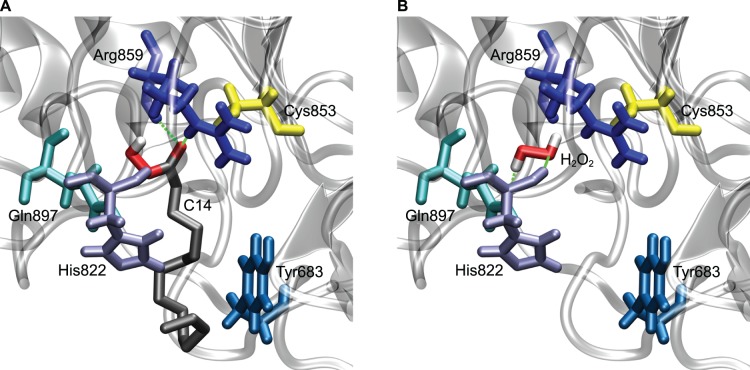
The best predicted binding poses of ligands in the CD45 active site. The best predicted binding poses for peroxy acid C14 (panel A) and hydrogen peroxide (panel B) in the CD45 active site. The receptor was based on the CD45 D1 domain from PDB structure 1YGU [20], with residues mutated to correspond to a CD45 reference sequence (UniProtKB accession number P08575) using the SWISS-MODEL web server [11]. The docking was performed with the AutoDock Vina version 1.1.1 software [15] using a rigid receptor and a binding box centered on the CD45 phosphatase active site. The best binding pose was defined as the pose with the strongest affinity in the largest cluster of poses, with poses clustered with a 1.5 Å RMSD thresholds. Also highlighted are four important residues involved in binding (Tyr683, His822, Arg859 and Gln897) and the catalytic cysteine (Cys853). Predicted hydrogen bonds with a 3.5 Å distance cutoff are shown as green dashed lines. The residues are numbered according the P08575 sequence.

The molecular docking computations determined the most likely binding poses of peroxytetradecanoic acid and hydrogen peroxide in the CD45 active site (Fig. 4). The analysis of the binding pose for peroxytetradecanoic acid (Fig. 4) shows that in the most likely binding pose, the ligand forms hydrogen bonds with Arg859; and also there are hydrophobic interactions between the acyl chain of peroxy acid and Tyr683 (residue positions numbered according to UniProtKB sequence P08575). These interactions contribute to the binding affinity calculated for this peroxy acid. The catalytic center of CD45 is highly positively charged [21] and may be an attractor for negatively charged peroxycarboxyl group. The docking computational analysis shows that peroxytetradecanoic acid is not sterically precluded from binding in the catalytic center of phosphatase CD45.

### Conclusions

We found that peroxytetradecanoic acid is a potent novel oxidative inhibitor of protein tyrosine phosphatase CD45, being effective in nanomolar concentrations. Moreover, the molecular docking results show that peroxytetradecanoic acid is not sterically excluded from binding to the CD45 active site, and furthermore that the increased binding affinity of peroxytetradecanoic acid relative to hydrogen peroxide could partially explain the stronger inhibitory effect observed in the experimental results.

Importantly, peroxydized tetradecanoic acid may be biosynthetically accessible via the reaction of endogenous hydrogen peroxide with myristic acid. Interestingly, this reaction may be catalyzed by lipases, which are abundance in the pancreatic acinar cells. The inactivation of protein tyrosine phosphatase CD45 expressed in the acinar cells due to peroxy acids formation may be implicated in the development and exacerbation of acute pancreatitis. The cytokine release associated with acute pancreatitis may be clarified by the report that CD45 (negative regulator of cytokines production) is down-regulated by redox-sensitive mechanisms in acinar cells during acute pancreatitis [7]. Based on colocalisation of lipase and protein tyrosine phosphatase CD45 in pancreatic cells, we suggest that peroxytetradecanoic acid being a potential lipase-catalyzed product might be an unexpected regulator of CD45 activity.

This is the first report on the effect of peroxytetradecanoic acid on the enzymatic activity of phosphatase CD45. The inhibitory properties of peroxytetradecanoic acid against protein tyrosine phosphatase were not previously described. Further studies on the impact of the shorter and longer acyl chain analogs of peroxy acids are underway.
